# Effects of Cyclic Thermal Stress at Later Age on Production Performance and Meat Quality of Fast-Growing, Medium-Growing and Thai Native Chickens

**DOI:** 10.3390/ani11123532

**Published:** 2021-12-11

**Authors:** Yuwares Malila, Anuwat Jandamuk, Thanawan Uopasai, Thongsa Buasook, Yanee Srimarut, Pornnicha Sanpinit, Yupin Phasuk, Sajee Kunhareang

**Affiliations:** 1 National Center for Genetic Engineering and Biotechnology (BIOTEC), Thailand Science Park, Tambon Khlong Nung 12120, Thailand; yanee.sri@biotec.or.th (Y.S.); pornnicha.san@ncr.nstda.or.th (P.S.); 2 Department of Animal Science, Faculty of Agriculture, Khon Kaen University, Khon Kaen 40002, Thailand; anuwat.ja@kkumail.com (A.J.); thanawanuopasai@kkumail.com (T.U.); yuplua@kku.ac.th (Y.P.); ksajee@kku.ac.th (S.K.); 3 Research Center and Central Laboratory, Faculty of Agriculture, Khon Kaen University, Khon Kaen 40002, Thailand; Thonbu@kku.ac.th

**Keywords:** chicken, carcass composition, growth performance, growth-related myopathies, meat quality, thermal stress

## Abstract

**Simple Summary:**

An increase in global surface temperature has raised a serious concern, as it can remarkably decrease broiler production, hence altering food security. This study investigated the effects of cyclic thermal stress, mimicking the rise of temperature during the day in tropical regions on production performance and meat quality of fast-growing commercial broilers (BRs), slow-growing Thai native (NT) chickens and medium-growing ones obtained from crossbreeding between BR and NT. The results indicated that, upon an exposure to thermal stress (35 ± 1 °C, 6 h daily) for 3 weeks prior to their specific market ages, all three chicken strains showed reduced final body weight and decreased average daily weight gain in comparison to their control counterparts. The adverse effects on production performance were less pronounced in the medium-growing and slow-growing chickens, with no significant alteration in their meat quality indices.

**Abstract:**

The present study aimed at assessing the impact of cyclic thermal stress on production performance and meat quality of commercial broilers (BRs), Thai native chickens (NT) and the hybrids between BR and NT (H75; crossbreed 25% NT). At the age of 3, 5 and 9 weeks for BR, H75 and NT, respectively, each strain was equally divided (*n* = 50) into control and treatment groups. The controls were raised at a constant 26 ± 1 °C, while the treatments were subjected to thermal stress (35 ± 1 °C, 6 h daily) for 3 weeks. The results indicated that final weight and average daily gain of BR and NT treated groups were significantly lower than those of their control counterparts. Reduced body weight gain of BR and H75, as well as feed intake of H75, was observed in the treatment groups (*p* < 0.05). The stressed BR breasts showed decreased moisture, fat and carbohydrate, accompanied by increased protein, ash, L *-value, b*-value and shear force (*p* < 0.05). No significant effects (*p* ≥ 0.05) of the thermal stress on meat quality indices were found for H75 and NT breast samples. Pectoral myopathies were observed in BR and H75 chickens, but the numbers of cases were decreased in the thermally treated groups.

## 1. Introduction

Driven by high consumer demand, commercial broilers have been intensively selected to achieve high production performance. The broilers undergo a markedly morphological and physiological transformation within the 6-week hatch-to-slaughter period. The birds, however, exhibit susceptibility to a variety of stresses, including thermal stress [1]. Modern broilers are more sensitive to thermal stress than their counterparts from 1950s and the ancestral wild jungle fowl [2], as broiler breeding was mainly focused on rapid growth and a heavy breast mass, but their respiratory and cardiovascular systems did not seem to develop at the same speed [3]. Their feathers work as thermal insulation, preventing heat dissipation, and their body parts that are not covered by feathers are in a smaller proportion. In this regard, when they encounter thermal stress, broilers reduce their feed intake and physical activities to minimize metabolic heat production, compromising body weight gain and overall broiler production performance [4,5,6,7,8,9]. Recent studies unraveled the events of disturbed metabolic homeostasis, altered antioxidant capacity and hormonal system and impaired gut integrity in the broilers exposed to experimental thermal stress condition [2,4,8,9]. In addition, properties of the meat, in terms of chemical composition, pH, water-holding capacity and texture, were deviated when the animals were exposed to heat stress [10,11,12]. The thermal sensitivity of the birds is higher with an increasing age and weight [13]. 

Thermal stress has been considered one of the most challenging factors of broiler production and has been discussed for decades [14]. Its impact is now even more concerning, due to the rise of average global surface temperature in the recent years [15]. In general, the broilers are raised under temperature-controlled housing facilities, such as evaporative cooling systems, to maintain the optimum temperature range. However, in tropical and subtropical regions, the facilities are not fully effective particularly on the days with an extreme temperature rise. In addition, such a management system requires high investment cost; therefore, it is not affordable for small-scale farmers [16]. Several strategies, such as nutrition intervention, have been shown to provide a beneficial effect against the thermal stress, with a varying degree of effectiveness [17,18,19,20]. Among the proposed mitigating approaches, the idea of developing the meat-type chicken lines with heat tolerance are considered as one of the effective long-term solutions. However, as heat tolerance and growth rate are negatively correlated, the genetic selection for both traits are still challenging [21].

In contrast to fast-growing commercial broilers, slow-growing native chickens pose an ability to withstand the harsh environment, including heat stress. This is because they are smaller in size and lighter in weight, and they have retained their adaptation to cope with the ambient temperature fluctuation [22]. Although slow-growing native chickens are less susceptible to the stress, such strains may not be the most effective alternative. As reviewed by Barbut [23], if one-third of the US broiler producers switched to a slower growing chicken breed, approximately 1.5 billion more birds would be raised annually to reach the similar amount of meat currently required. Additional resources, e.g., feed, water, fuel and land, would certainly be consumed. Crossbreeding between the native slow-growing chickens and commercial broilers could introduce heat tolerant traits from the native line to the commercial lines [20,24,25,26]. Therefore, the objective of this study was to compare growth performance, carcass composition and meat quality indices of the three genetic lines of chickens, namely the commercial broiler (BR), the Thai native Chee (NT) and the crossbreed between BR and NT, upon an exposure to cyclic thermal stress during the three weeks prior to their specific market ages.

## 2. Materials and Methods 

### 2.1. Animals and Management

The experiment was set up at the Department of Animal Science, Faculty of Agriculture, Khon Kaen University (Thailand). A total of 300 chicks (100 chicks/strain, mixed male and female), representing three genetic lines, including BR (Ross 308), NT (100% Thai native Chee) and H75 (crossbred; 75% BR and 25% NT), were used in this study. One-day-old Ross 308 chicks were obtained from a local hatchery (Charoen Pokphand Company, Nakhon Ratchasima, Thailand). The chicks of NT and the crossbreeds were obtained from the Department of Animal Science, Faculty of Agriculture, Khon Kaen University (Khon Kaen, Thailand). Upon receiving, the chicks were individually tagged with wing bands and assigned in floor pens (1.3 m × 2.0 m). The chicks were raised under an environmentally controlled poultry facility, strictly following the standard practice for commercial meat-type chickens. Rice hulls were used as bedding material. Water and feed were provided ad libitum. All birds were fed the same standard commercial broiler diets, including starting phase (21% crude protein, 3100 kcal of ME/kg, 5% crude fiber, 13% moisture and 4% fat) for 1 day to 3 weeks of age and growing phase (20% crude protein, 3200 kcal of ME/kg, 5% crude fiber, 13% moisture and 4% fat) thereafter until the birds were slaughtered. A routine vaccination program against coccidiosis, infectious bronchitis virus and Newcastle disease was applied.

When the BR, H75 and NT reached the age of 3, 5 and 9 weeks, respectively, the birds from each strain were randomly divided into two equal groups, i.e., control (*n* = 50) and treatment (*n* = 50), with an equal body weight and placed into two separated houses. The control groups (4 replications/group, 12 to 13 birds/replication, 5 birds/m^2^) were raised under a constant temperature of 26 ± 1 °C, controlled by air conditioners. For the treatment groups (4 replications/group, 12 to 13 birds/replication, 5 birds/m^2^), the temperature was raised from 26 ± 1 °C to 35 ± 1 °C during 10:00 a.m. to 4:00 p.m. (6 h daily) to mimic the rise of the ambient environment during the day of the local region and reduced to approximately 26 ± 1 °C afterwards. The temperature in the thermally stressed house was also controlled by using air conditioners assisted with heat radiators. Apart from the different temperature profiles, the control and treatment samples were reared in a similar manner. The birds were closely monitored throughout the experimental period of 3 weeks. Feed intake (FI) and the mortality were recorded daily. The feed-to-gain ratio (F/G) was calculated as FI divided by BWG after the correction for mortality.

### 2.2. Sample Collection

Upon completion of the 3-week experimental period, the BR, H75 and NT chickens reached their market age at 6, 8 and 12 weeks of age, respectively. All of the birds were then proceeded to the slaughtering process. Before sample collection, the birds were fasted for 12 h. The birds were individually weighed. Slaughtering process included a manual neck cut, bleeding for 3 min, scalding at 60 °C for 1 min and placement in a rotary drum picker for 30 s. Weight of each whole hot carcass was recorded. The carcasses were immediately advanced to evisceration and sectioned into parts. Visceral organs, breasts, thighs, livers and abdominal fat were weighed. Left sides of the breasts from each bird were individually packed in polyethylene bags and stored at 4 °C, until reaching 24-h postmortem for meat-quality determination. 

### 2.3. Meat Quality Determination

Upon 24-hour postmortem, all breast samples from each group were inspected for growth-related myopathies, including white striping (WS) and wooden breast (WB). The inspection was carried out by one trained individual, following the classification criteria described by Kuttappan et al. [27] and Tijare et al. [28] for WS and WB, respectively. In brief, the samples showing any white lines on the meat surface were classified as WS, whereas the meat with hard ridges was classified as WB. After the assessment of WS and WB, 24 breasts from each treatment were randomly selected: 12 breasts were used for the determination of pH and chemical composition, whereas the remaining 12 breasts of each treatment were used for the analyses of surface color, drip loss, cook loss, total processing loss and texture.

Evaluation of pH was carried out by directly inserting a spear-shaped probe equipped with a pH meter (Mettler-Toledo Seven Easy, Mettler-Toledo, Inc., Greifensee, Switzerland) into three assigned positions (cranial, medial and caudal) of each raw meat sample. 

The chemical compositions, including moisture, protein, lipid and ash, of breast samples were determined by following the Association of Official Analytical Chemists (AOAC) standard methods [29]. In brief, each breast sample was thoroughly ground prior to the analyses. Moisture content was determined based on weight loss upon drying the samples in a conventional oven (model FD115, Binder GmbH, Tuttlingen, Germany) at 100 ± 2 °C for 16 h. Protein content was measured by following a Dumas combustion method, using a Dumatherm (model DUMATHERM N Pro, C. Gerhardt GmbH & Co. KG, Königswinter, Germany). Lipid content was determined based on a petroleum ether extraction method, using the Soxhlet method (Soxtherm, model SOX416, C. Gerhardt GmbH & Co. KG, Königswinter, Germany). Ash was assessed based on an incineration of the samples at 600 °C. Proximate composition for each sample was carried out in triplicates.

Surface color, drip loss, cook loss and texture of cooked breast samples were examined consecutively in the same piece of the meat according to the method described by U-chupaj et al. [30] with a slight modification. Briefly, the meat samples were bloomed on ice for 30 min, and the surface color of the meat in the CIE L*, a*, b* system was determined by using a handheld colorimeter (model WR-18, Shenzhen Graigar Technology Co., Ltd., Baoan District, China). The values of ∆E, indicating color difference between control and treatment within the same chicken strain, were then calculated by using the following equation: ∆E = √[(L*_control_ − L*_treatment_)^2^ + (a*_control_ − a*_treatment_)^2^ + (b*_control_ − b*_treatment_)^2^].

Subsequently, the meat was weighed, wrapped with a couple of layers of gauze cloth and hung at 4 °C for 24 h. The meat was gently dapped dry with a paper towel and reweighed. Drip loss was expressed in percentage as the weight difference before and after hanging. The meat was subsequently cooked at 95 °C by water immersion until the core temperature of the thickest part of the breast reached 80 °C [30]. The cooked meat was then cooled down in running tap water, and left to rest at 4 °C for 2 h before reweighed. Cook loss (%) was expressed as the weight difference before and after cooking. Total processing loss (%) indicated difference in weight of the meat prior to drip loss determination and after being cooked. The cooked meat was then cut into 1 cm × 1 cm × 1 cm cubes for Texture Profile Analysis (TPA), and into 1 cm × 2 cm × 1 cm specimens for Warner Braztler Shear (WBS) test. During the TPA and WBS analysis, the meat was either double-compressed or cut perpendicular against muscle fiber alignment, using a texture analyzer (model TA.HDplusC, Stable Micro Systems, Goldalming, UK) equipped with a 50 mm cylindrical aluminum probe or a V-slot WB Blade, respectively. All textural parameters were automatically calculated and reported by the Exponent software (Stable Micro Systems, Goldalming, UK). 

### 2.4. Statistical Analyses

Statistical analysis was performed by using the R package version 3.2.1. Mean difference between control and thermally treated groups within the same chicken strains was analyzed using Student’s *t*-test, except for mortality data, which were analyzed using the Chi-square test. Significant level for all statistical analyses was set at α = 0.05.

## 3. Results

### 3.1. Growth Performances

The effects of thermal stress during the last three weeks of age on growth performance of the chickens are shown in Table 1. As for BR chickens, thermal stress significantly reduced final BW, BWG and ADG (*p* < 0.05). Similar to the BR, the BWG, ADG and FI of the hybrid H75 were decreased upon exposure to the stress (*p* < 0.05). The NT chickens exposed to thermal stress also exhibited lower values of final body weight and ADG (*p* < 0.05), but the BWG and FI between control and stressed NT groups were not significantly different. As for F/G, although the values were trending upward when the birds were exposed to cyclic thermal stress, no significant differences (*p* ≥ 0.05) between control and treatment groups were found in the chicken strains. Concerning mortality rate of the BR breed, the results showed that control and stressed samples exhibited a similar mortality rate at 8%. Thermally stressed H75 showed a mortality rate at 2%, whereas the control group of this chicken line showed no loss. As for the NT chickens, there was no loss in either control or treatment.

### 3.2. Carcass Composition

The adverse effects of thermal stress on carcass composition did not show statistical significance (Table 2). It is worth noting that no abdominal fat was found in the NT chickens either from control or treatment group.

### 3.3. Meat Quality

#### 3.3.1. Chemical Composition

The effects of thermal stress on chemical composition between control and thermally treated groups of each chicken line are shown in Table 3. Breasts of the thermally stressed BR samples showed decreased moisture, fat and carbohydrate, along with increased protein and ash, compared to their control counterparts (*p* < 0.05). No significant changes in the chemical composition of breast samples were observed in H75 chickens. As for NT chickens, a significant (*p* < 0.05) increase in fat content was found in the stressed samples. 

#### 3.3.2. Surface Color

According to Table 4, the effects of thermal stress on surface color of the raw chicken breasts were observed only in the BR samples (*p* < 0.05). Those BR samples exposed to thermal challenge exhibited an increased L*-value and b*-value, indicating that the breasts appeared lighter and more yellow than those of the control samples. Based on an ∆E value of 3.2, the color difference between the control and treatment of the broilers was likely visually detected by consumers [31].

#### 3.3.3. pH, Water-Holding Capacity and Texture

Concerning meat quality indices, as shown in Table 5, the thermal condition used in this study did not significantly impact the values of pH, drip loss, cook loss and total processing loss of the breast samples from all chicken lines (*p* ≥ 0.05). As for the texture (Table 6) of the cooked breast samples, only the BR samples showed a significant difference between the control and thermally treated groups (*p* < 0.05). The shear force and shear energy of the BR samples exposed to thermal stress were higher than those of their control counterparts (*p* < 0.05), while no significant changes were observed for the TPA parameters (*p* ≥ 0.05).

#### 3.3.4. Cases of Growth-Related Myopathies

**Figure 1:**
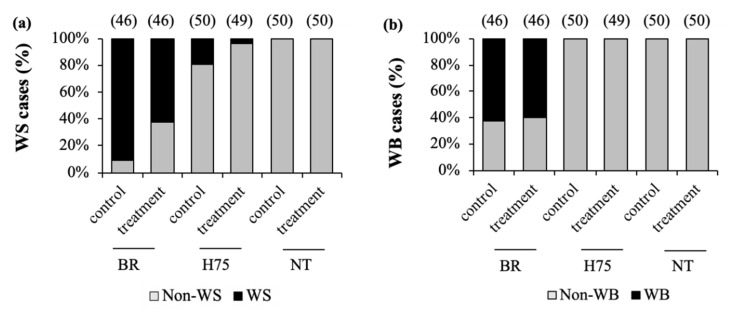
Incidences (%) of (**a**) white striping (WS) and (**b**) wooden breast (WB) abnormalities among three different strains of chickens raised without (control) or with (treatment) thermal stress at the last three weeks of age. BR = commercial broiler; H75 = crossbreed 75% BR and 25% NT; NT = Thai native Chee. Numbers in parentheses above bars indicate number of the samples examined for the abnormalities.

Focusing on the cases of growth-related myopathies (Figure 1), as expected, WS and WB cases were the most pronounced in BR groups, of which the control showed more cases of both WS (control = 91%, treatment = 63%) and WB abnormalities (control = 62%, treatment = 59%) compared to the stressed BRs. For H75 chickens, WS cases were found at 19% and 4% of the control and of the stressed H75 groups, respectively, whereas no H75 birds exhibited WB abnormality. Neither WS nor WB was found in NT samples.

## 4. Discussion

In this study, the effects of an exposure to cyclic thermal stress, mimicking the daily rise of temperature in the local area, on performance and breast-meat quality were investigated among the three strains of chickens, representing fast-growing commercial broilers, slow-growing Thai NT and medium-growing crossbreeds between those two strains. The reduced growth performance found in all chicken strains, particularly BR, was consistent with previous studies [6,7,11,13,32,33]. Physiological response and the outcome appeared to be strain-dependent [7,11]. Several studies showed that, for commercial broilers, the reduction of BW upon exposure to a high environmental temperature was related with decreased feed intake modulated by the increased serum corticosterone [32,34]. This stress hormone plays essential roles in various vital physiological responses to stimuli. Upon thermal challenges, corticosterone induced insulin resistance. Together, they activated fat synthesis, resulting in fat deposition in the stressed animals [34]. The growth performance of the locally slow-growing chickens, such as Beijing You chicken [11] and Thai NT Chee [25,35], and medium-growing chickens [25] was diminished when the birds were facing thermal challenge, but those strains showed a greater tolerance to high ambient temperature than the broilers.

In this study, the lack of significant changes in FI and F/G ratio between BR control and treatment groups could be attributed to less intensity of thermal challenge compared to previous studies. Xie et al. [4] assigned Abor Acres broilers into control and treatment groups with the temperature set at constant 21 and 33 °C (8 weeks), respectively. Duangjinda et al. [33] compared the growth performance of broilers and Thai NT chickens under either thermoneutral (26 °C) or chronic thermal stress (36 to 38 °C, 6 h daily) for three weeks and reported the thermal-induced suppression of growth performance for BR but not in the NT chickens. In the study of Emami et al. [9], broilers were exposed to 35 ± 1 °C for 8 h during the age of 29 to 42 days, while the control broilers were kept at 23 °C. Shao et al. [36] applied a similar thermal condition to that used in the study of Emami et al. [9] and reported significant decreases in body weight gain and feed intake in Chinese yellow-feather broilers exposed to the challenge. Souza et al. [5] showed that nutrient utilization and energy metabolism of broilers were not affected by a cyclic thermal exposure (32 °C, 8 h daily). Based on a recent meta-analysis by Andretta et al. [13], the feed conversion ratio was not significantly affected by heat stress. 

In addition, it could be speculated that the stressed BR samples could partly exhibit thermal acclimatization. In agreement with our hypothesis, Aengwanich [25] demonstrated that a ratio of heterophil and lymphocyte (H/L ratio), the biomarker indicating heat stress for chickens, of broilers was spiked after 7 days of thermal exposure but despite the greater levels than those observed in Thai native crossbreeds and Thai native chickens, the levels of the marker were reduced afterwards. When exposed to cyclic thermal stress, the broilers older than 21 days were sometimes able to compensate their feed intake during the non-stress period [13]. Furthermore, Lu et al. [34] reported that the respiratory rate of broilers exposed to thermal challenge (32 °C) for 14 days was not different from the control group, suggesting the heat acclimatization or heat tolerance in the later period of the stress. Xie et al. [4] reported that, among broiler breeders, prolonged thermal stress resulted in tissue damage identified as increased levels of plasma lactate dehydrogenase, glutamic-oxaloacetic transaminase and creatine phosphokinase; however, it did not disturb feed intake, the plasma metabolites (i.e., glucose, triglyceride, total protein uric acid and cholesterol), stress hormones (i.e., corticosterone and thyroid hormones) and plasma peroxidation indicators (i.e., malonaldehyde and superoxide dismutase). 

Although the current cyclic thermal condition did not apparently affect carcass composition and meat yield of the BR samples, the properties of the breast meat, the most valuable part of the BR chickens, were visually and texturally altered. The current findings agreed with previous studies [7,10,11,37,38]. Heat stress impacted the meat quality of the chickens through oxidative damages, which further modulated postmortem metabolism, reducing the pH of the muscle and meat [7,39]. Under such an unusual acidic condition, meat protein could undergo severe denaturation and excessively lose the ability to retain water, leading to low water-holding capacity of the meat. Myoglobin, red water-soluble pigment in meat, was potentially lost with the drips; therefore, the meat became paler with the increasing L*-value [39,40]. In addition, an accumulation of reactive oxygen species upon receiving the challenge may contribute to oxidation of proteins, emphasizing the changes in color, water-holding capacity and texture of the meat [39]. However, in this study, changes in the pH and water-holding capacity of the meat between control and treatment groups were not significant (*p* ≥ 0.05), and this agreed well with some previous reports [11,36,38]. Zhang et al. [38] and Shao et al. [36] found that the cyclic thermal challenge did not exert significant impacts on lactic acid content either in chicken breast or thigh meat. Together, the results supported the potential acclimatization of the birds to the current cyclic thermal condition [4]. For the BR birds, however, the oxidative damage might have already occurred and affected the color and texture of the BR meat. Further investigation of oxidative status is underway to test the speculation.

Concerning chemical composition of breast meat, only the BR samples showed the differences between control and treatment groups. However, the current trend was somewhat in the opposite direction from other previous studies [10,38], showing reduced protein content due to suppressed protein synthesis and enhanced proteolysis [41] in heat-stressed samples. However, the current results, underlying the greater moisture and fat, along with the lower protein in the control BR, were well explained by the greater incidence of WS and WB, the two important emerging growth-related myopathies in broilers, in the control BR breast samples than their counterparts [42,43]. The actual etiology of the myopathies is under an extensive investigation but growing evidence pointed out the relation between the abnormalities and the selection for high production performance of fast-growing broilers [23,42]. The lower WS and WB cases in the treated BR and H75 samples could be attributed to suppressed growth performance under the current thermal challenge. In the case of the H75 crossbreeds, development of WS could be due to the genetic influence from the BRs. Nevertheless, all WS samples could be graded as mild level (white line thickness < 1 mm). The present findings demonstrated that, similar to BRs, the growth performance of the medium-growing H75 and NT chickens was suppressed; however, the quality of the breast meats of NT and H75 chickens were not affected by the current cycle thermal challenge. The results were as expected, as previous studies have shown that native chickens pose a better capability to adapt and tolerate against thermal challenge [20,24,25,26]. Although the H75 chickens required two more weeks, compared to the BR, to reach their market age with the lower breast and thigh yields, their performance and the yield were superior to those of the NT chickens. In addition, the H75 chickens exhibited greater thermal tolerance under the current challenge than the BR. The findings suggested the potential benefits of this strain for small-scale farmers in the region.

## 5. Conclusions

Overall, the outcome evidenced that the current cyclic thermal-stress condition adversely affected the growth performance of all three strains of the tested chickens, but the effect was less pronounced in H75 and the NT chickens. The BRs appeared to exhibit thermal adaption under an exposure of the current cyclic thermal stress, but the visual color and texture of their breast meat were significantly impaired. The chemical composition of the BR breasts was influenced by the occurrence of growth-related myopathies. The meat of NT and H75 chickens was not affected by the current cycle thermal challenge. 

## Figures and Tables

**Table 1:** Growth performance of the three different strains of chickens, as affected by thermal stress on the last three weeks of age.

Strains	Thermal Stress	Initial BW (g)	Final BW (g)	BWG (%)	ADG (g/d)	FI (g/d)	F/G	Mortality (%)
BR	Control	519.8 ± 91.0	1740.5 ± 283.8	238.8 ± 50.1	57.4 ± 12.3	99.5 ± 27.0	1.7 ± 0.2	8
	Treatment	539.0 ± 80.9	1620.4 ± 231.5	203.9 ± 41.3	51.2 ± 9.6	93.6 ± 26.6	1.8 ± 0.1	8
	*p*-value ^1^	0.31	0.04	0.001	0.01	0.14	0.45	0.96
H75	Control	671.5 ± 133.3	1428.3 ± 277.2	114.0 ± 23.4	36.0 ± 8.3	85.0 ± 20.0	2.4 ± 0.3	0
	Treatment	666.3 ± 122.1	1346.6 ± 281.8	102.2 ± 22.6	32.4 ± 9.0	77.2 ± 28.6	2.6 ± 0.7	2
	*p*-value	0.85	0.17	0.02	0.05	0.04	0.62	0.56
NT	Control	745.2 ± 148.6	1131.4 ± 191.2	54.1 ± 18.4	18.4 ± 4.5	60.9 ± 14.6	3.3 ± 0.5	0
	Treatment	684.2 ± 198.5	902.3 ± 261.3	47.7 ± 21.3	14.7 ± 4.8	61.5 ± 15.0	4.2 ± 0.8	0
	*p*-value	0.18	0.02	0.22	0.003	0.83	0.16	1.00

^1^ Comparisons between the control and treatment groups (*n* = 50) within the same strain based on Student’s *t*-test, except for mortality that Chi-square test was applied. Statistical significance was set at *p* < 0.05. BR = commercial broiler; H75 = crossbreed 75% BR and 25% NT; NT = Thai native Chee; BW = body weight; BWG = body weight gain; ADG = average daily gain; FI = feed intake, F/G = feed-to-gain ratio.

**Table 2:** Carcass composition of the three different strains of chickens, as affected by thermal stress on the last three weeks of age.

Strains	Thermal Stress	Whole Carcass (g)	Visceral Organs (%)	Breast (%)	Thigh (%)	Abdominal Fat (%)	Liver (%)
BR	Control (*n* = 46)	1526.8 ± 283.2	12.7 ± 2.4	24.3 ± 2.8	10.3 ± 0.7	1.7 ± 0.7	2.5 ± 0.4
	Treatment (*n* = 46)	1472.5 ± 215.8	12.9 ± 1.7	23.8 ± 2.8	10.5 ± 0.7	1.6 ± 0.9	2.4 ± 0.4
	*p*-value ^1^	0.31	0.66	0.46	0.23	0.41	0.36
H75	Control (*n* = 50)	1267.3 ± 234.7	13.6 ± 2.5	17.4 ± 2.0	10.7 ± 1.2	2.2 ± 1.2	2.3 ± 0.4
	Treatment (*n* = 49)	1218.9 ± 224.4	13.8 ± 2.8	16.8 ± 2.1	11.0 ± 0.7	2.3 ± 1.1	2.3 ± 0.3
	*p*-value	0.33	0.72	0.15	0.26	0.96	0.85
NT	Control (*n* = 50)	993.0 ± 176.8	13.8 ± 2.5	15.2 ± 1.9	12.1 ± 1.6	nd	2.2 ± 0.3
	Treatment (*n* = 50)	920.5 ± 226.9	13.8 ± 2.4	14.9 ± 1.8	11.5 ± 0.8	nd	2.1 ± 0.3
	*p*-value	0.18	0.93	0.51	0.12	na	0.44

^1^ Comparisons between the control and treatment groups within the same strain based on Student’s *t*-test. Statistical significance was set at *p* < 0.05. BR = commercial broiler; H75 = crossbreed 75% BR and 25% NT; NT = Thai native Chee; nd = not detected; na = not applicable.

**Table 3:** Chemical composition of breast meat collected from the three different strains of chickens, as affected by thermal stress on the last three weeks of age.

Strains	Thermal Stress	Moisture (%)	Protein (%)	Fat (%)	Carbohydrate (%)	Ash (%)
BR	Control	75.73 ± 0.43	21.31 ± 0.46	0.99 ± 0.17	0.75 ± 0.25	1.22 ± 0.09
	Treatment	74.92 ± 0.61	22.43 ± 0.52	0.78 ± 0.17	0.46 ± 0.24	1.41 ± 0.11
	*p*-value ^1^	0.001	<0.001	0.004	0.02	0.01
H75	Control	74.42 ± 1.28	22.60 ± 1.07	0.70 ± 0.21	0.57 ± 0.30	1.76 ± 0.24
	Treatment	74.24 ± 0.69	22.67 ± 0.69	0.60 ± 0.18	0.85 ± 0.35	1.63 ± 0.25
	*p*-value	0.66	0.84	0.29	0.05	0.20
NT	Control	73.15 ± 0.49	23.96 ± 0.62	0.30 ± 0.07	0.65 ± 0.33	1.94 ± 0.06
	Treatment	73.37 ± 0.24	23.64 ± 0.47	0.40 ± 0.12	0.67 ± 0.30	1.85 ± 0.08
	*p*-value	0.23	0.23	0.002	0.25	0.05

^1^ Comparisons between the control and treatment groups (*n* = 12) within the same strain based on Student’s *t*-test. Statistical significance was set at *p* < 0.05. BR = commercial broiler; H75 = crossbreed 75% BR and 25% NT; NT = Thai native Chee.

**Table 4:** Surface color of raw breast meat collected from the three different strains of chickens, as affected by thermal stress on the last three weeks of age.

Strains	Thermal Stress	L*-Value	a*-Value	b*-Value	ΔE ^2^
BR	Control	40.87 ± 3.38	–1.15 ± 0.68	1.18 ± 1.12	3.23
	Treatment	43.76 ± 2.31	–1.11 ± 0.50	2.63 ± 1.74	
	*p*-value ^1^	0.02	0.86	0.03	
H75	Control	43.18 ± 3.74	–0.68 ± 0.86	1.99 ± 1.83	0.56
	Treatment	43.29 ± 2.54	–1.13 ± 0.42	1.67 ± 2.06	
	*p*-value	0.94	0.13	0.69	
NT	Control	44.05 ± 2.45	–0.97 ± 0.40	1.51 ± 1.29	0.71
	Treatment	44.72 ± 2.60	–1.15 ± 0.55	1.64 ± 1.32	
	*p*-value	0.58	0.42	0.83	

^1^ Comparisons between the control and treatment groups (*n* = 12) within the same strain based on Student’s *t*-test. Statistical significance was set at *p* < 0.05. ^2^ ∆E = √[(L*_control_ − L*_treatment_)^2^ + (a*_control_ − a*_treatment_)^2^ + (b*_control_ − b*_treatment_)^2^]. The value indicates the difference in visual surface color of raw meat between control and treatment within the same strain. BR = commercial broiler; H75 = crossbreed 75% BR and 25% NT; NT = Thai native Chee.

**Table 5:** Values of pH of breast meat collected from the three different strains of chickens, as affected by thermal stress on the last three weeks of age.

Strains	Thermal Stress	pH	Drip Loss (%)	Cook Loss (%)	Total Processing Loss (%)
BR	Control	6.01 ± 0.10	1.67 ± 0.53	14.64 ± 1.83	16.07 ± 2.00
	Treatment	5.97 ± 0.13	1.29 ± 0.53	13.85 ± 2.75	14.96 ± 2.87
	*p*-value ^1^	0.92	0.09	0.41	0.28
H75	Control	5.81 ± 0.08	2.25 ± 0.52	15.20 ± 3.70	17.20 ± 3.57
	Treatment	5.84 ± 0.09	2.48 ± 0.78	17.15 ± 2.91	19.20 ± 3.14
	*p*-value	0.29	0.40	0.18	0.16
NT	Control	5.77 ± 0.11	2.72 ± 0.79	15.20 ± 3.47	17.50 ± 3.60
	Treatment	5.78 ± 0.82	3.15 ± 0.84	15.68 ± 1.64	18.33 ± 2.23
	*p*-value	0.82	0.28	0.71	0.57

^1^ Comparisons between the control and treatment groups (*n* = 12) within the same strain based on Student’s *t*-test. Statistical significance was set at *p* < 0.05. BR = commercial broiler; H75 = crossbreed 75% BR and 25% NT; NT = Thai native Chee.

**Table 6:** Textural characteristics of cooked breast meat collected from the three different strains of chickens, as affected by thermal stress on the last three weeks of age.

Strains	Thermal Stress	Shear Force (N)	Shear Energy (N.s)	Hardness (N)	Springiness	Cohesiveness	Chewiness (N)
BR	Control	24.4 ± 7.0	100.6 ± 26.2	10.5 ± 1.9	0.59 ± 0.04	0.57 ± 0.04	3.6 ± 0.7
	Treatment	30.1 ± 8.9	121.3 ± 38.5	10.7 ± 2.8	0.60 ± 0.03	0.60 ± 0.03	4.0 ± 0.6
	*p*-value ^1^	0.04	0.04	0.77	0.28	0.05	0.10
H75	Control	20.4 ± 10.1	87.2 ± 42.5	8.8 ± 1.1	0.63 ± 0.03	0.57 ± 0.05	3.2 ± 0.7
	Treatment	29.8 ± 16.6	122.5 ± 60.9	8.7 ± 2.0	0.61 ± 0.03	0.58 ± 0.04	3.1 ± 0.9
	*p*-value	0.08	0.11	0.89	0.19	0.39	0.64
NT	Control	29.1 ± 14.0	122.2 ± 52.3	8.3 ± 1.5	0.62 ± 0.03	0.61 ± 0.02	3.2 ± 0.5
	Treatment	26.6 ± 10.6	121.7 ± 36.9	8.0 ± 2.1	0.62 ± 0.02	0.59 ± 0.03	3.1 ± 0.8
	*p*-value	0.68	0.98	0.74	0.71	0.24	0.63

^1^ Comparisons between the control and treatment groups (*n* = 12) within the same strain based on Student’s *t*-test. Statistical significance was set at *p* < 0.05. BR = commercial broiler; H75 = crossbreed 75% BR and 25% NT; NT = Thai native Chee.

## Data Availability

The data presented in this study are available on request from the corresponding author.
